# Influence of drainage divides versus arid corridors on genetic structure and demography of a widespread freshwater turtle, *Emydura macquarii krefftii*, from Australia

**DOI:** 10.1002/ece3.968

**Published:** 2014-02-11

**Authors:** Erica V Todd, David Blair, Dean R Jerry

**Affiliations:** 1 School of Marine and Tropical Biology, James Cook UniversityTownsville, Queensland, 4810, Australia; 2 Centre for Tropical Water and Aquatic Ecosystem Research, James Cook UniversityTownsville, Queensland, 4810, Australia; 3 Centre for Sustainable Tropical Fisheries and Aquaculture, James Cook UniversityTownsville, Queensland, 4810, Australia

**Keywords:** Burdekin Gap, Chelidae, cyclic aridity, drainage divide, phylogeography, population genetics

## Abstract

The influence of Pleistocene climatic cycles on Southern Hemisphere biotas is not yet well understood. Australia's eastern coastal margin provides an ideal setting for examining the relative influence of landscape development, sea level fluctuation, and cyclic climatic aridity on the evolution of freshwater biodiversity. We examined the impact of climatic oscillations and physical biogeographic barriers on the evolutionary history of the wide-ranging Krefft's river turtle (*Emydura macquarii krefftii*), using range-wide sampling (649 individuals representing 18 locations across 11 drainages) and analysis of mitochondrial sequences (∼1.3-kb control region and *ND4*) and nuclear microsatellites (12 polymorphic loci). A range of phylogeographic (haplotype networks, molecular dating), demographic (neutrality tests, mismatch distributions), and population genetic analyses (pairwise *F*_ST_, analysis of molecular variance, Bayesian clustering analysis) were implemented to differentiate between competing demographic (local persistence vs. range expansion) and biogeographic (arid corridor vs. drainage divide) scenarios. Genetic data reveal population genetic structure in Krefft's river turtles primarily reflects isolation across drainage divides. Striking north-south regional divergence (2.2% *ND4 p*-distance; *c*. 4.73 Ma, 95% higher posterior density (HPD) 2.08–8.16 Ma) was consistent with long-term isolation across a major drainage divide, not an adjacent arid corridor. Ancient divergence among regional lineages implies persistence of northern Krefft's populations despite the recurrent phases of severe local aridity, but with very low contemporary genetic diversity. Stable demography and high levels of genetic diversity are inferred for southern populations, where aridity was less extreme. Range-wide genetic structure in Krefft's river turtles reflects contemporary and historical drainage architecture, although regional differences in the extent of Plio–Pleistocene climatic aridity may be reflected in current levels of genetic diversity.

## Introduction

Climatic cycles over the past several million years have driven important evolutionary changes in the world's biota. However, research has focused largely on the Northern Hemisphere where land-based glaciation was extensive. Widespread glaciations never occurred in Australia, which instead experienced cycles of severe aridity that also profoundly influenced current patterns of biodiversity (Byrne et al. 2008, 2011). Extreme hydrological variability also accompanied these cycles (Kershaw et al. 2003), yet evolutionary responses of freshwater species to such cyclic aridity are not yet well understood. Understanding how evolutionary histories of freshwater species are tied to the underlying biotic and geological evolution of a region is significant, because their dispersal depends upon direct hydrological connectivity within and across drainage divides and because the history of drainage interconnectivity reflects historical influences of landscape, sea level, and climate (Bermingham and Avise 1986; Bernatchez and Wilson 1998; Waters et al. 2001).

Within an otherwise largely arid and geologically quiescent continent, Australia's narrow eastern margin still supports numerous permanent drainages and a diverse endemic freshwater fauna. Volcanism, erosion, scarp retreat, and sea level fluctuations have shaped complex drainage patterns (Chappell et al. 1996; Jones 2006; Vasconcelos et al. 2008) among a series of coastal-flowing systems formed by Cenozoic uplift of the Great Dividing Range (GDR). The GDR parallels the entire length of Australia's east coast, separates coastal and interior drainages, and shelters the eastern margin from widespread aridity. In recent years, phylogeographic studies of fishes and macroinvertebrates have highlighted the biogeographic complexity of the eastern margin, revealing cryptic diversity among individual drainage basins and deep lineage disjunctions at higher geographic scales (Wong et al. 2004; Jerry 2008; Page et al. 2012).

Lowlands within the geographically extensive Burdekin drainage (130,400 km^2^) currently experience the most severe seasonal aridity of anywhere along Australia's east coast. The degree of aridity experienced here was probably even more extreme during glacial maxima (Williams 1984). The “Burdekin Gap” arid corridor is a well-recognized vicariant barrier driving north-south divergence in many terrestrial animal lineages (Chapple et al. 2011), and its influence dates to the mid-to-late-Miocene or Pliocene (James and Moritz 2000; Moussalli et al. 2005). More recently, molecular studies of several wide-ranging freshwater taxa also report deep north-south lineage disjunction congruent with the Gap (e.g., freshwater fishes, Wong et al. 2004; Jerry 2008; Unmack and Dowling 2010; and platypus, a freshwater mammal, Gongora et al. 2012). However, the Burdekin drainage hosts substantially lower species diversity of freshwater fishes compared with adjacent drainages, and inadequate sampling has so far precluded accurate assessment of possible alternative historical barriers to freshwater dispersal. Genetic breaks observed in previous studies are also consistent with a major drainage divide between the Burdekin and the equally extensive Fitzroy basin (141,100 km^2^), immediately to its south. These basins may be particularly ancient (Jones 2006), and bathymetric data suggest they maintained independent paleochannels during glacial low stands despite a broad continental shelf (∼160 km wide; Fielding et al. 2003; Ryan et al. 2007). It remains unclear whether north-south genetic disjunction in Australia's eastern freshwater fauna reflects climatic aridity, as for terrestrial taxa, or a physical drainage divide. Australia's eastern margin presents an excellent opportunity to examine the relative importance of climate versus drainage topography in influencing patterns of freshwater biodiversity.

Freshwater and terrestrial turtles have proven sensitive phylogeographic models for inferring how historical evolutionary forces shape observable patterns of intraspecific molecular diversity (Walker and Avise 1998; Weisrock and Janzen 2000), but remain poorly studied outside the Northern Hemisphere. Australian freshwater turtles (Chelidae: Pleurodira) are part of the continent's original Gondwanan fauna. They represent underutilized model taxa for uncovering regional biogeographic patterns and for inferring underlying evolutionary forces driving Australia's unique freshwater biodiversity. Species of the genus *Emydura* are especially common throughout eastern Australia. They have broad opportunistic diets and generalist habitat requirements, and although primarily riverine, occupy a variety of permanent freshwater habitats from rivers to billabongs. Electrophoretic surveys have revealed *Emydura* species to be very closely related genetically and presumably represent a recent radiation (Georges and Adams 1996). Their apparently shallow evolutionary history may imply an ecological capacity to survive climatic perturbation and to exploit opportunities during favorable conditions.

**Figure 1:**
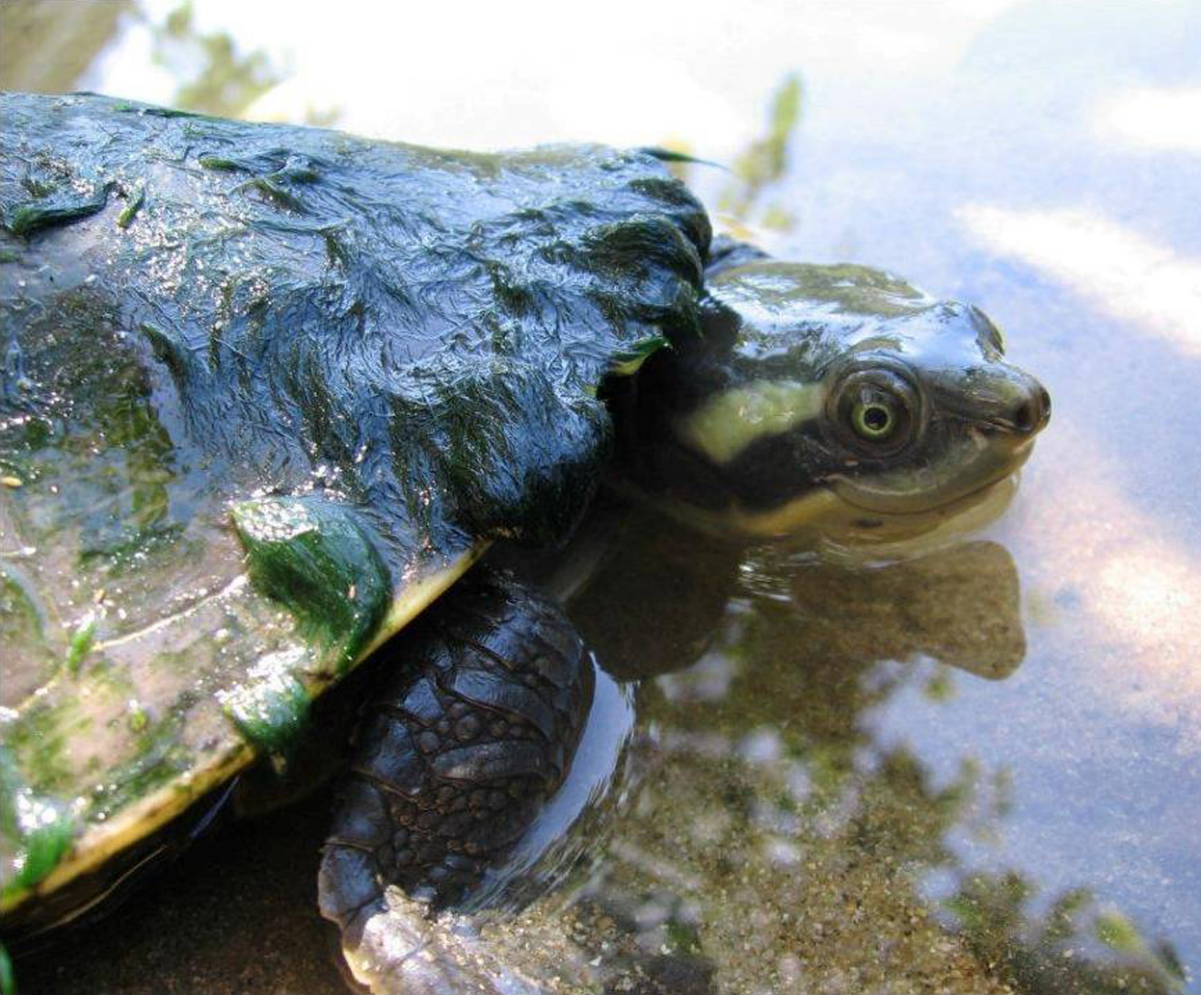
The study organism, Krefft's river turtle *Emydura macquarii krefftii*, collected from the Pioneer River in eastern Australia. Photo by Erica Todd.

**Figure 2:**
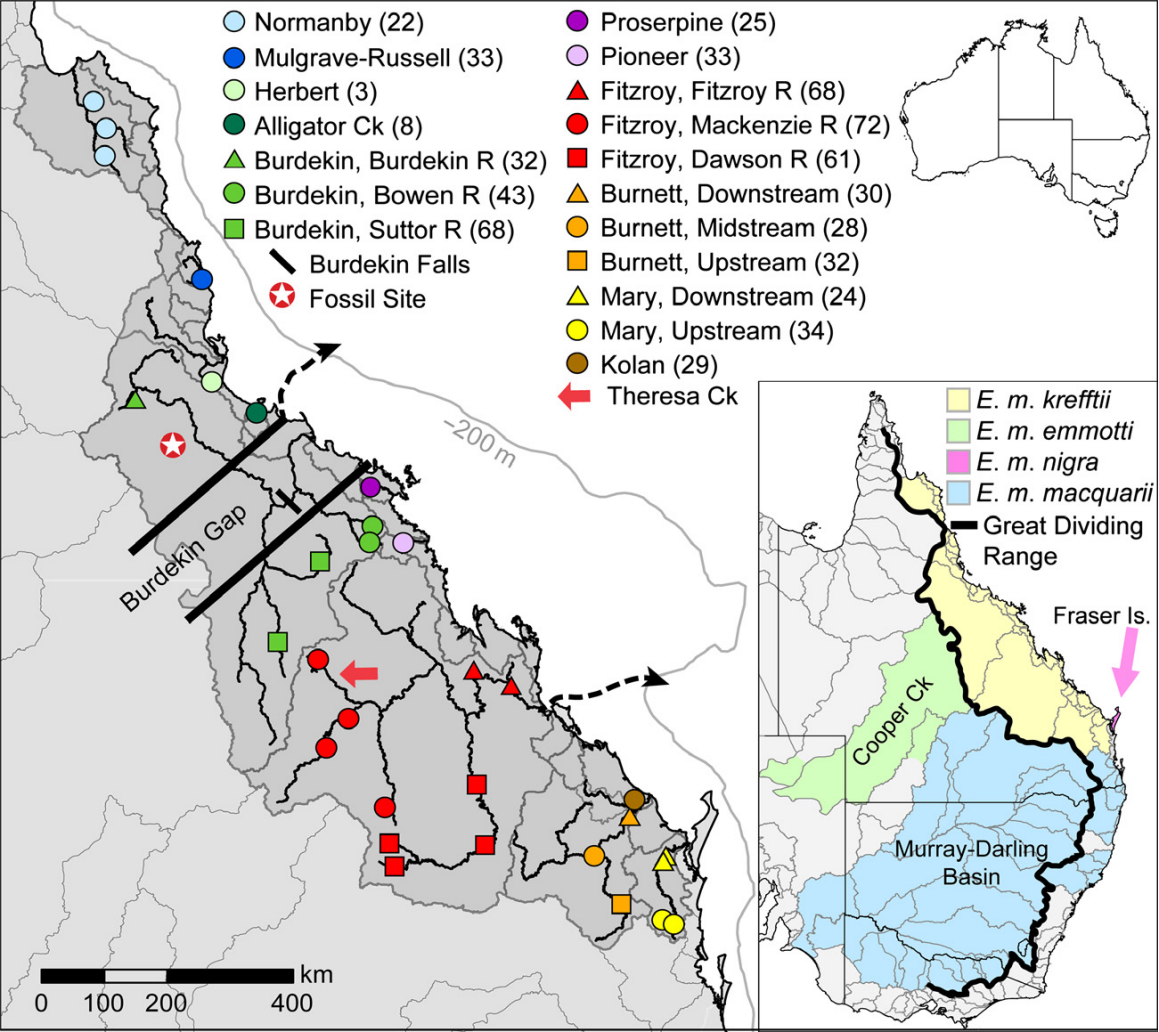
Map showing the distribution of *Emydura macquarii* subspecies (inset) and sampling locations (left) for *Emydura macquarii krefftii* across 11 drainages in eastern Australia. Samples are grouped by drainage (color), then subregion (shape, where appropriate). Samples sizes are given in brackets. Drainage boundaries are outlined in dark gray. The distribution of *E*. *m*. *krefftii* is in darker gray, and river networks are in black for drainages where samples were collected. Dashed arrows indicate likely palaeochannels of the Fitzroy (Ryan et al. 2007) and Burdekin (Fielding et al. 2003) Rivers, indicating independent trajectories to the continental shelf, indicated in light gray at the −200 m contour. Important features mentioned in text are indicated, including the Burdekin Gap, Burdekin Falls, Bluff Downs fossil site, and Theresa Ck.

Krefft's river turtle, *E*. *m*. *krefftii* (Fig. 1), is one of four subspecies within the southern *Emydura macquarii* complex, currently defined largely by nonoverlapping geographical ranges (Fig. 2) and minor morphological differences in facial coloration and body shape/size (Georges and Thomson 2010). Krefft's turtle is ideally suited to investigating patterns of genetic differentiation, as it occupies an extensive longitudinal distribution east of the GDR that includes major regions of coastal aridity, as well as wetter regions to the north and south. It is one of few freshwater species, and the only turtle species, found throughout the arid Burdekin drainage. Although its response to historical aridity in this region is unknown, fossil evidence places it within the Burdekin drainage in the early Pliocene (Thomson and Mackness 1999), prior to initiation of Pleistocene phases of aridity. However, whether Krefft's turtles survived glacial aridity in this region within isolated local refugia – experiencing bottlenecks and latter demographic expansions, or whether current populations reflect northwards range expansion from a southern stronghold – for example, since the most recent aridification, is yet to be determined.

To examine the relative importance of a major drainage divide versus a historical arid corridor as barriers to movement of freshwater animals in eastern Australia, we investigated range-wide genetic structure in Krefft's river turtle, *E*. *m*. *krefftii*. A combination of mitochondrial sequences and nuclear microsatellite loci were used to: (1) describe population genetic structure and connectivity in *E*. *m*. *krefftii* at multiple spatial scales, including within and among individual drainage basins as well as regionally across its whole distribution; and (2) differentiate between competing biogeographic (arid corridor vs. drainage divide) and demographic (local persistence vs. range expansion) hypotheses. If the Burdekin Gap has presented an important historical barrier for Krefft's turtles, regional genetic divergence is expected among populations either side of the Burdekin lowlands. Alternatively, if the Burdekin–Fitzroy drainage divide has been a significant historical barrier, regional genetic divergence is expected across the Burdekin's southern drainage boundary. Similarly, if *E*. *m*. *krefftii* survived local climatic perturbation in northern refugia, (a) northern populations are expected to be genetically differentiated from southern populations; and (b) to potentially show signals of historical bottlenecks and/or recent demographic expansion. By contrast, if northern populations are the result of recent range expansions from southern refugia, we would expect (a) steady northwards decrease in genetic diversity; and (b) less pronounced genetic structure moving northwards. In either case, southern populations are expected to have been demographically more stable.

## Materials and Methods

### Sample collection and DNA extraction

Tissue samples were obtained from 649 *E*. *m*. *krefftii* across 18 locations in 11 drainage basins, representing the native distribution of this subspecies in eastern coastal Australia (Fig. 2; Additional File 1). A hierarchical sampling design allowed for examination of population connectivity and genetic structure at three spatial scales: local river (among locations within drainages), drainage basin (among drainages within regions), and regional (whole distribution). To investigate the role of specific biogeographic features in structuring turtle populations, samples were especially sourced from across the Burdekin Gap dry corridor within the Burdekin drainage, and the Burdekin–Fitzroy drainage divide, including geographically intermediate, peripheral coastal streams (Proserpine and Pioneer Rivers; Fig. 2). Samples were also obtained from five representatives of the other three *Emydura macquarii* subspecies (*Emydura macquarii macquarii*, Murray–Darling drainage; *Emydura macquarii emmotti*, Cooper Ck drainage; *Emydura macquarii nigra*, Lake Birrabeen, Fraser Is; Additional File 1) for comparison. Tissues were obtained from existing collections through collaborations with other researchers, or during targeted field work. In the field, turtles were caught by seine net, baited trap or by hand while snorkeling. A small section of skin was removed from the forelimb of each turtle and preserved immediately in 95% ethanol, later stored at −20°C. Genomic DNA was extracted from tissues using a modified salting-out protocol described previously (Todd et al. 2013a).

### Genetic structure and diversity at mitochondrial DNA

We investigated historical population structure and identified major genetic lineages within *E*. *m*. *krefftii* by sequencing ∼1.3 kb of the mitochondrial genome. Turtle-specific primers were used to amplify sections of the hypervariable control region (CR; 439 bp) and NADH dehydrogenase subunit 4 (*ND4*; 670 bp) using primers and protocols described previously (Todd et al. 2013a). Sequences were aligned and edited using GENEIOUS PRO 5.6 (Biomatters, available from http://www.geneious.com). *ND4* sequences were translated and examined for premature stop codons that may indicate the presence of nuclear mitochondrial (numt) DNA paralogues, and sequence chromatograms were inspected visually for ambiguous nucleotide signals that may indicate co-amplification of mitochondrial DNA and numts. Finally, regions were aligned with published sequences from a range of turtle species to verify amplification of the correct mitochondrial region. No evidence of numts was found, and CR and *ND4* sequences were concatenated for further analysis.

Relatedness and spatial distribution of mtDNA haplotypes (*E*. *m*. *krefftii* and related subspecies) were assessed with a minimum spanning network, computed in ARLEQUIN 3.5 (pairwise distance model; Excoffier and Lischer 2010). The minimum spanning tree was drawn for clarity of presentation.

Measures of mtDNA diversity within major *E*. *m*. *krefftii* lineages and overall were calculated in ARLEQUIN and DNASP 5.10 (Librado and Rozas 2009), including number of haplotypes (*N*_h_), number of polymorphic sites (*N*_p_), haplotype diversity (*h*), nucleotide diversity (*π*), and average number of nucleotide differences (*k*).

Evolutionary distances within and among major genetic lineages of *E*. *m*. *krefftii*, as well as among related subspecies, were estimated for *ND4* sequences using mean uncorrected *p*-distances in MEGA 5 (Tamura et al. 2011), with pairwise deletion of sites containing gaps and using 1000 bootstrap replicates for variance estimation. Three individuals geographically located within one lineage but carrying a haplotype from another (i.e., suggestive of recent dispersal) were excluded in order to gain accurate estimates of lineage divergence. Calculations were based on *ND4* only as a common measure of genetic divergence that will be comparable across studies of other reptiles and fishes (Fritz et al. 2012).

### Genetic structure and diversity at microsatellite markers

To assess contemporary genetic structure and diversity, individuals were genotyped at 12 polymorphic microsatellite loci designed specifically for *E*. *m*. *krefftii* (Ekref04, Ekref06-Ekref10, Ekref12-Ekref15, Ekref18, and Ekref20; Todd et al. 2011). Details of multiplex PCR conditions, primer sequences and fragment analysis are presented in Todd et al. (2011). Tests for conformation to Hardy–Weinberg expectations were performed for each locus in GENALEX 6.5 (Peakall and Smouse 2006, 2012). Potential linkage disequilibrium between pairs of loci was examined in GENEPOP 4.0 (10,000 permutations; Rousset 2008), and the presence of null alleles was evaluated using MICRO-CHECKER 2.2.3 (Van Oosterhout et al. 2004). Significance levels for multiple comparisons were adjusted using a false discovery rate (FDR) correction (Benjamini and Hochberg 1995).

Genetic diversity was assessed by calculating number of alleles (*N*_A_) and observed (*H*_O_) and expected (*H*_E_) heterozygosities for each sample location, drainage and region in ARLEQUIN. Private alleles were identified using GENALEX. Allelic richness (*A*_R_) was also calculated for each population, region and overall in FSTAT 2.9 (Goudet 1995) because this statistic corrects for sample size variation. A hierarchical analysis of allelic richness was also performed in FSTAT (1000 permutations) to test for differences in allelic richness among regional groups identified in preliminary analyses.

Population genetic structure within and across drainage boundaries was quantified using pairwise linearized *F*_ST_ values, calculated among sampling locations and drainages. For the large Burdekin and Fitzroy drainages, locations were pooled by subcatchment. Analyses were implemented in FSTAT, with significance levels adjusted for multiple comparisons. Hierarchical population genetic structure across the *E*. *m*. *krefftii* distribution was examined using analysis of molecular variance (AMOVA), performed in ARLEQUIN (10,000 permutations). AMOVA were performed at two spatial scales: regional (among northern and southern regions and among drainages within regions) and drainage (among drainages and among locations within drainages).

An individual-based Bayesian clustering approach, performed in the program STRUCTURE 2.3 (Pritchard et al. 2000), was used to identify the number of genetically homogeneous groups (*K*) best represented within the microsatellite data. All samples were utilized, including those from the Herbert (*n* = 2) and Alligator Ck (*n* = 8) drainages. Ten independent simulations were run for each value of *K* from one to 10, using 5 million MCMC replications with an initial “burn-in” of 1 million, found to be appropriate in pilot runs. An admixture model with correlated allele frequencies was assumed, and sampling locations were used as prior information (LOCPRIOR model), which improves clustering performance for datasets where the signal of genetic structure may be weak, without biasing the outcome (Hubisz et al. 2009). Summary statistics (log likelihood and α) were monitored for each run to verify convergence. The true value of *K* was determined following the posterior probability (Pritchard et al. 2000) and delta log likelihood methods (Evanno et al. 2005), implemented in STRUCTURE HARVESTER 0.6 (Earl and von Holdt 2012). To test for further substructure within major genetic units and reconstruct potential hierarchical relationships among clusters, each identified cluster was subsequently run independently. For the chosen *K* value in each analysis, average pairwise similarity (*H’*) of STRUCTURE runs was assessed in the program CLUMPP 1.1.2 (Jakobsson and Rosenberg 2007), using the Greedy algorithm with 10,000 random additions on the 10 independent runs. Results were imported into DISTRUCT 1.1 (Rosenberg 2004) for graphical representation.

### Bayesian analysis of recent migration

**Figure 3:**
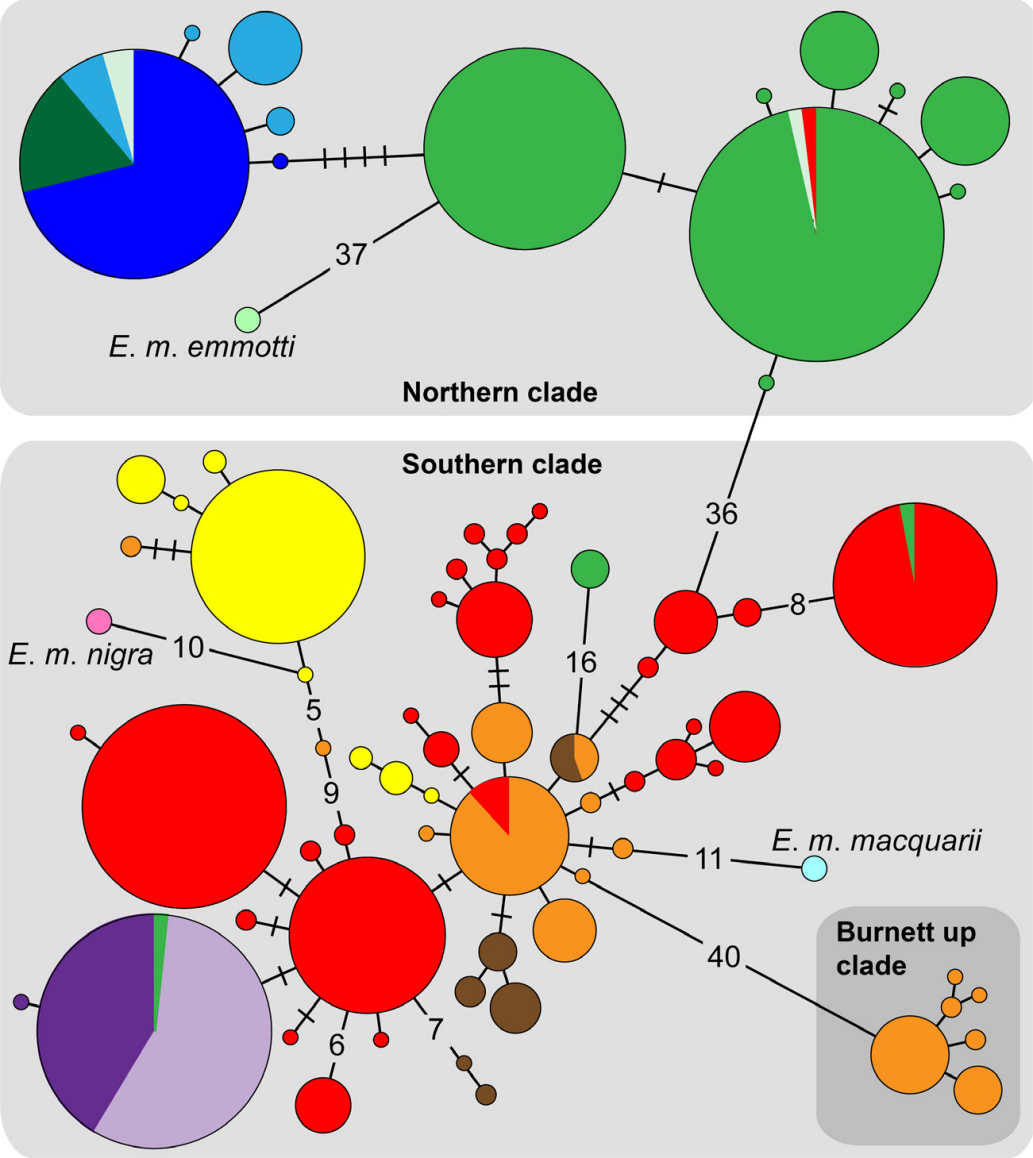
Minimum spanning tree depicting relationships among mtDNA haplotypes for concatenated control region and *ND4* sequences from *Emydura macquarii krefftii* across 11 river drainages. Haplotypes sampled from related subspecies *Emydura macquarii macquarii* (pale blue), *Emydura macquarii nigra* (pale pink), and *Emydura macquarii emmotti* (pale green) are also included. Circles represent unique haplotypes and are connected to one another by mutational changes (lines). Cross-bars indicate additional mutational changes between haplotypes, which are represented numerically when there are many. Circle size is proportional to haplotype frequency and colors represent sampling location (drainages) following Figure 2.

Initial analyses of mitochondrial and microsatellite data revealed two genetically divergent lineages within *E*. *m*. *krefftii* separated across the Burdekin–Fitzroy drainage divide, with evidence of recent admixture across this barrier (see Results and Figs. 3, 5). To investigate this further, recent migration (last few generations) across the drainage divide was quantified using a Bayesian modeling approach in BAYESASS 3.0.3 (Wilson and Rannala 2003). BAYESASS estimates unidirectional migration rates (*m*) for each population using individual multilocus microsatellite data, on the basis that recent immigrants and their offspring display genotypic disequilibrium relative to the population in which they were sampled. The approach does not assume migration–drift equilibrium, but because prior limits are placed on *m* (0–0.33), is powerful only when migration is low (*m *<* *0.1) and population divergence is moderate–high (*F*_ST_ ≥ 0.05; Faubet et al. 2007).

Samples were pooled according to sample location north or south of the Burdekin–Fitzroy drainage divide. In pilot runs, mixing parameters were adjusted to achieve acceptance rates between 20% and 40%. Convergence was assessed using TRACER 1.5 (available from http://beast.bio.ed.ac.uk./Tracer). Based on recommendations by Faubet et al. (2007), five replicate analyses of 22 × 10^6^ iterations with 2 × 10^6^ “burn-in”, sampling every 2000 steps, were performed using different seeds to ensure trace convergence and consistency of results. The command-g was used to produce estimates of individual migrant ancestries.

### Divergence time estimation between northern and southern mitochondrial lineages

A timeframe for divergence between northern and southern mitochondrial lineages (Fig. 3) was estimated in BEAST 1.8.0 (Drummond et al. 2012). Individuals suspected to be recent migrants (three individuals geographically located within one lineage but carrying a haplotype from another) were excluded. Concatenated mtDNA sequences were analyzed using a HKY+I+G model of sequence evolution, as selected by jMODELTEST 0.1.1 (Guindon and Gascuel 2003; Posada 2008) under the BIC criterion. Analyses assumed a strict molecular clock, with a rate prior normally distributed around the mean of reptilian mtDNA divergence rates of 0.47–1.32% per million years (Zamudio and Greene 1997), scaled per lineage per million years (mean 4.5 × 10^−3^, SD 1.1 × 10^−3^). This approach follows that of Hodges et al. (2014), who successfully applied this rate in a divergence analysis of another Australian chelid, *Chelodina expansa*. Given the intraspecific nature of the current dataset, a coalescent tree prior (constant population size) was applied. Two independent MCMC chains were each run for 200 million generations, sampling every 20,000 steps. Trace convergence was assessed in TRACER and replicate runs combined using LOGCOMBINER 1.8.0 (10% “burn-in”). TREEANNOTATOR 1.8.0 was used to produce the maximum clade credibility tree, visualized in FIGTREE 1.3.1 (available from http://tree.bio.ed.ac.uk/software/figtree).

### Tests of demographic history: mitochondrial and microsatellite data

Neutrality statistics and mismatch distributions were performed on mtDNA data to test for signatures of population expansion within major *E*. *m*. *krefftii* clades. Fu's *F*_S_ (Fu 1997) is one of the most powerful neutrality tests for detecting population expansion and was calculated along with Tajima's *D* (Tajima 1989) in ARLEQUIN, with 10,000 coalescent simulations to assess significance. Neutrality statistics are sensitive to historical demography, selection, and/or population structure. Significant negative values indicate population expansion or purifying selection, while significant positive values indicate population bottleneck, balancing selection, or admixture. The hypothesis of recent population expansion was evaluated by calculating the mismatch distribution of pairwise differences between haplotypes (Rogers and Harpending 1992) against a model of sudden population expansion in ARLEQUIN. A smooth or unimodal distribution signifies recent population expansion, whereas a ragged or multimodal distribution represents demographic equilibrium or decline (Rogers and Harpending 1992; Excoffier 2004). To test whether the data matched a sudden expansion model, sum of squared deviations (SSD) and raggedness indices (*r*) were calculated, using 2000 bootstrap replicates to assess significance. Significant SSD or *r* values indicate that an expansion model is rejected by the data.

Microsatellite data were used to investigate the possibility of recent genetic bottlenecks using tests for heterozygosity excess implemented in BOTTLENECK 1.2.02 (Cornuet and Luikart 1997; Piry et al. 1999). Populations that have recently undergone significant reduction in size are expected to exhibit heterozygosity excess at polymorphic loci as allele numbers are reduced faster than heterozygosity due to rapid loss of rare alleles (Cornuet and Luikart 1997). Samples were grouped by region (north and south) as well as according to intraregional groups identified in STRUCTURE analyses. Tests were conducted under a two-phase model (TPM), which incorporates elements of the infinite allele model (IAM) and stepwise mutation model (SMM; variance = 12, SMM = 80%, 85%, 90% and 95%, Piry et al. 1999). Statistical significance was tested with a Wilcoxon's signed-rank test (10,000 permutations), considered most robust for datasets of <20 polymorphic loci (Piry et al. 1999). Significance levels were adjusted for multiple tests using FDR as above.

## Results

### Mitochondrial DNA diversity and historical population structure

Among 646 individuals sequenced at both CR and *ND4*, a total of 72 unique haplotypes were identified, encompassing 121 variable sites across a 1276 bp concatenated alignment. Three individuals failed to amplify at both loci and were excluded from the concatenated dataset. For CR only, 56 haplotypes were identified among 649 individuals (62 variable sites across 439 bp) [GenBank: KF181795–KF181853]. Twenty-nine *ND4* haplotypes were identified among 647 individuals (59 variable sites across 837 bp) [GenBank: KF181854–KF181885]. The haplotypic network, based on concatenated sequences, distinguished three divergent clades within *E*. *m*. *krefftii* (Fig. 3). Two main clades, separated by 37 mutational changes, were geographically delineated by the Burdekin–Fitzroy drainage divide. Turtles from the Pioneer and Proserpine Rivers, geographically intermediate between the Burdekin and Fitzroy drainages, belong within the southern lineage. There were three instances of haplotype sharing across the Burdekin–Fitzroy boundary. Three individuals sampled close to the drainage divide possessed a haplotype from the opposite lineage, suggesting recent dispersal across the watershed in both directions (Fig. 3). A single haplotype, sampled from six individuals from the upstream Suttor R (Burdekin drainage), was also nested within the southern lineage, but distinguished by 17 mutations. Few haplotypes were shared among drainages, except in the following circumstances: a single common haplotype was shared by the adjacent Pioneer and Proserpine Rivers, and another was shared across the four most northerly drainages excluding the Burdekin. However, in the southern clade, haplotypes unique to particular drainages did not necessarily cluster together. A central (potentially ancestral) haplotype occurs in the Burnett and Fitzroy Rivers. A cluster of haplotypes from the Mary River is distinct by several mutations. Within the northern clade, subdivision occurs between the large Burdekin drainage and elsewhere. A third divergent clade within *E*. *m*. *krefftii* consists of six closely related haplotypes sampled exclusively from a location in the upstream Burnett River, separated by 41 mutations from other Burnett R haplotypes.

For the remaining subspecies, only a single haplotype was identified per subspecies among the five sampled individuals. Their relationships with *E*. *m*. *krefftii* haplotypes are also shown in Figure 3. Haplotypes from the southernmost subspecies, *E*. *m*. *macquarii* (Murray–Darling Basin) and *E*. *m*. *nigra* (Fraser Is), were nested within the southern *E*. *m*. *krefftii* clade and were closely related to haplotypes from the Burnett and Mary Rivers, respectively. *E*. *m*. *emmotti* (Cooper Ck) was distinct, separated by 38 mutational changes from the northern *E*. *m*. *krefftii* clade. *ND4* evolutionary distances are presented in Table 1 and show divergence among *E*. *m*. *krefftii* clades (2.23–3.03%) to be considerably greater than that between the southern *E*. *m*. *krefftii* clade and *E*. *m*. *nigra* (0.83%) or *E*. *m*. *macquarii* (0.64%). The upstream Burnett clade was as divergent from the southern clade (2.31%) as from the other subspecies (range 2.35–2.83%), giving no indication of a potential historical relationship. Greatest within-clade divergence was seen within the southern clade (0.53%), compared with the northern (0.19%) or upstream Burnett (0.16%) clades.

**Table 1:** Mean evolutionary distances within and among major *Emydura macquarii krefftii* lineages and *Emydura macquarii* subspecies based on uncorrected *ND4 p*-distances (% divergence). Lower matrix, percentage divergence among lineages/subspecies; upper matrix, SE values for percent divergence among lineages/subspecies; bold diagonal, percentage divergence within lineages/subspecies.

	*Emk* “N”	*Emk* “S”	*Emk* “BU”	*Emm*	*Emn*	*Eme*
*Emk* “Northern”	**0.19**	0.46	0.56	0.48	0.49	0.44
*Emk* “Southern”	2.23	**0.53**	0.46	0.20	0.25	0.45
*Emk* “Burnett Up”	3.03	2.31	**0.16**	0.49	0.50	0.55
*Emm*	2.06	0.64	2.35	–	0.28	0.49
*Emn*	2.23	0.83	2.51	0.72	–	0.50
*Eme*	1.63	2.01	2.83	1.91	2.15	–

*Emk*,*E*. *m*. *krefftii*;*Emm*,*Emydura macquarii macquarii*;*Emn*,*Emydura macquarii nigra*;*Eme*,*Emydura macquarii emmotti*.

Measures of mtDNA diversity are presented in Table 2. The northern clade was divided into Burdekin and Far North subclades as per the haplotype network. Greatest genetic diversity was found within the southern clade (68 haplotypes, *H*_d_ = 0.940, *π *= 0.662). The northern clade shared just three common haplotypes across 200 individuals (*H*_d_ = 0.812, *π *= 0.325; Table 2).

**Table 2:** Measures of mitochondrial DNA diversity (1276 bp, concatenated control region and *ND4*), neutrality tests and demographic parameters within three major clades and two subclades of Krefft's river turtle *Emydura macquarii krefftii*. Results of the observed mismatch distribution against a sudden expansion model include the raggedness index (*r*) and the SSD.

	Northern	Northern: far north	Northern: Burdekin	Southern	Burnett up	Overall
*N*	200	65	135	412	30	642
*N*_p_	17	4	9	68	5	121
*N*_h_	13	5	8	53	6	72
*H*_d_ (SD)	0.812 (0.012)	0.477 (0.062)	0.706 (0.022)	0.940 (0.005)	0.685 (0.067)	0.957 (0.002)
*π* (SD)	0.325 (0.180)	0.041 (0.039)	0.108 (0.074)	0.662 (0.339)	0.076 (0.059)	1.971 (0.958)
*k* (SD)	4.140 (2.069)	0.552 (0.444)	1.37 (0.85)	8.42 (3.91)	0.96 (0.67)	25.15 (11.05)
*D*	1.440	−1.399*	−0.167	−0.862	−0.726	na
*F*_S_	2.066	−1.662	−0.551	−8.710	−1.761	na
*r*	0.039	0.161	0.059	0.018	0.118	na
SSD	0.029	0.013	0.016	0.011	0.010	na

*N*, sample size; *N*_p_, number of polymorphic sites; *N*_h_, number of haplotypes; *H*_d_, haplotype diversity; *π*, nucleotide diversity (expressed as percentages, i.e., 0.001 = 0.1%); *k*, average number of nucleotide differences; SD, standard deviation; *F*_S_, Fu's statistic; *D*, Tajima's *D*-test; *r*, raggedness index; SSD, sum of squared deviations; na, not applicable.

Significant tests are indicated with asterisks as follows:

* *P *<* *0.05.

### Microsatellite diversity and contemporary population structure

A total of 128 alleles were identified across the 12 microsatellite loci within 644 Krefft's river turtles with <5% missing data (5–17 alleles per locus). Deviations from HWE were detected at six loci (Ekref04, Pioneer R and Burdekin Suttor R; Ekref18, Burdekin Suttor R; Ekref20, Normanby R; Ekref07, Mary downstream; Ekref12, Mary upstream; Ekref09, Fitzroy Dawson R, and Fitzroy Mackenzie R). There was no evidence of scoring error due to stuttering or large allele dropout at any locus, although tests for homozygote excess in MICRO-CHECKER suggested null alleles at locus Ekref15 and Ekref12 in the Fitzroy R subcatchment and Normanby R populations, respectively. There was no evidence for linkage disequilibrium. As there was no consistent evidence for null alleles or deviation from HWE for any particular locus or sampling location, and as analyses with and without these data did not alter the interpretation, the entire dataset was retained and examined.

As for mtDNA, stark differences were found in measures of genetic diversity among regions and some sampling locations at microsatellites (Table 3). Values of genetic diversity were considerably and consistently lower within the northern clade compared with the southern clade, both overall and when averaged across sampling locations (Table 3). The only exception was the Pioneer and Proserpine Rivers within the southern clade, which showed similarly low levels of genetic diversity as recorded for northern locations. Differences in *A*_R_, *H*_O_, and gene diversity among regions were all statistically significant (*P *<* *0.05). For the southern locations collectively, 48 (40%) private alleles were detected, compared with eight (10%) in the northern locations. Most individual drainages also contained private alleles (range: 0 in Proserpine R, Mulgrave–Russell R and Normanby R to 8 in Fitzroy R and Burdekin R).

**Table 3:** Microsatellite diversity (12 loci) across sampling locations (≥10 sample size) for Krefft's river turtle (*Emydura macquarii krefftii*). Values are given for each catchment, region and overall (bold), with averages (italics) across multiple sampling locations where appropriate.

Drainage (no. of sample locations)	Sample size (±SD)	Mean No. alleles (±SD)	Private alleles	*H*_E_ (±SD)	*H*_O_ (±SD)	*A*_R_^1^ (±SD)
Mary	58	7.33 (3.08)	4	0.66 (0.20)	0.66 (0.20)	6.39 (2.00)
*Average* (2)	*29 (7.1)*	*6.63 (0.53)*		*0.66 (0.01)*	*0.66 (0.01)*	*6.31 (0.22)*
Burnett	90	7.25 (2.73)	2	0.69 (0.14)	0.68 (0.13)	6.06 (2.05)
*Average* (3)	*30 (2.0)*	*6.14 (0.24)*		*0.69 (0.02)*	*0.68 (0.04)*	*5.85 (0.15)*
Kolan	29	5.08 (2.06)	1	0.65 (0.16)	0.68 (0.19)	4.99 (1.95)
Fitzroy	201	8.42 (3.50)	8	0.70 (0.21)	0.69 (0.22)	6.40 (2.44)
*Average* (3)	*67.0 (5.6)*	*7.50 (0.14)*		*0.70 (0.01)*	*0.69 (0.01)*	*6.32 (0.08)*
Pioneer	33	4.75 (2.22)	2	0.49 (0.24)	0.50 (0.25)	4.35 (1.99)
Proserpine	25	3.25 (1.54)	0	0.44 (0.26)	0.48 (0.26)	3.19 (1.48)
**South**	**436**	**10.00 (3.67)**	**48**	**0.71 (0.18)**	**0.65 (0.17)**	**9.48 (3.50)**
***Average*** **(11)**	***39.6 (18.0)***	***6.11 (1.34)***		***0.64 (0.09)***	***0.64 (0.08)***	***5.61 (1.03)***
Burdekin	143	6.17 (3.54)	8	0.53 (0.27)	0.51 (0.27)	4.49 (2.57)
*Average* (3)	*47.7 (18.4)*	*4.83 (0.47)*		*0.53 (0.03)*	*0.53 (0.05)*	*4.26 (0.19)*
M-Russell	33	3.50 (2.19)	0	0.46 (0.28)	0.57 (0.21)	3.25 (1.90)
Normanby	22	2.58 (1.83)	0	0.30 (0.23)	0.30 (0.23)	2.58 (1.83)
**North**^2^	**208**	**6.67 (4.16)**	**8**	**0.53 (0.26)**	**0.48 (0.25)**	**6.67 (4.16)**
***Average*** **(5)**	***34.3 (20.3)***	***4.06 (0.98)***		***0.47 (0.09)***	***0.50 (0.11)***	***3.72 (0.78)***
**Overall**^2^	**644**	**10.67 (4.01)**		**0.70 (0.14)**	**0.60 (0.15)**	**9.69 (3.56)**

*H*_E_, expected heterozygosity; *H*_O_, observed heterozygosity; *A*_R_, allelic richness; SD, standard deviation.

1 *A*_R_ standardized to min. sample size of 22 for location and catchment, 208 for region and overall.

2 Includes samples from Alligator Ck (*n* = 8) and Herbert R (*n* = 2).

Microsatellites revealed a considerable level of genetic differentiation across the range of *E*. *m*. *krefftii*. Pairwise *F*_ST_ values among drainages were all highly significant after correction for multiple comparisons (Table 4). However, within discreet drainage basins, *F*_ST_ values among sampling locations were low (<0.016; mean *F*_ST_ 0.008, SD 0.005), suggesting drainage basins are the most local unit of population structure. Only three comparisons were significant after correction: Burnett downstream vs. upstream (*F*_ST_ 0.008), Burdekin Bowen R vs. Suttor R (*F*_ST_ 0.016), and Burdekin Bowen R vs. Burdekin R (*F*_ST_ 0.015). Distinctiveness of the upstream Burnett R population, as supported by mtDNA (Fig. 3), was not strongly supported by the nuclear data. This location was therefore included in all analyses of microsatellite data. Pairwise comparisons among drainages were also highest across regions (mean *F*_ST_ 0.274, SD 0.090), compared with within regions (mean *F*_ST_ 0.144, SD 0.074). AMOVA analyses also revealed significant hierarchical population structure (Table 5). At a regional scale, 10.4% of genetic variation was attributed to variation among regions (north and south; *P *=* *0.012), and 9.9% to variation among drainages within regions (*P *=* *0.000). A drainage-level analysis attributed 15.9% of genetic variation to among drainages (*P *=* *0.000), with a small but significant 0.7% (*P *=* *0.000) attributed to among locations within drainages.

**Table 4:** Pairwise *F*_ST_ comparisons between drainage basins for Krefft's river turtle, *Emydura macquarii krefftii*, based on 12 polymorphic microsatellite markers. Bold values are comparisons across “northern” and “southern” geographic regions. All values are significant at 0.001 after correction for multiple comparisons.

Drainage	Mary	Burn	Kolan	Fitzr	Pion	Pros	Burd	MR	Norm
Mary	–								
Burnett	0.038	–							
Kolan	0.060	0.044	–						
Fitzroy	0.076	0.059	0.100	–					
Pioneer	0.187	0.150	0.215	0.087	–				
Proserpine	0.226	0.204	0.237	0.148	0.186	–			
Burdekin	**0.161**	**0.171**	**0.212**	**0.173**	**0.240**	**0.294**	–		
M-Russell	**0.216**	**0.223**	**0.269**	**0.199**	**0.270**	**0.330**	0.114	–	
Normanby	**0.307**	**0.304**	**0.334**	**0.291**	**0.457**	**0.484**	0.198	0.272	–

**Table 5:** Hierarchical analysis of molecular variance (AMOVA) results for *Emydura macquarii krefftii* microsatellite data analyzed at two spatial scales.

Source of variation	Variation (%)	Fixation index	*P*-value
Regional scale			
Among regions	10.42	*F*_CT_ = 0.104	0.012
Among drainages within regions	9.93	*F*_SC_ = 0.111	0.000
Within drainages	79.64	*F*_ST_ = 0.204	0.000
Drainage scale			
Among drainages	15.87	*F*_CT_ = 0.159	0.000
Among locations within drainages	0.73	*F*_SC_ = 0.009	0.000
Within locations	83.39	*F*_ST_ = 0.167	0.000

Analyses in STRUCTURE further highlighted hierarchical population structure in *E*. *m*. *krefftii*. A primary division between two genetic clusters (highest delta *K* for *K *=* *2; Fig. 5A) corresponded to northern and southern mtDNA clades and the Burdekin–Fitzroy drainage divide. However, distinct regions of admixture were evident in the Pioneer and Proserpine Rivers and a group of six individuals sampled from the upstream Fitzroy drainage (Theresa Ck, Mackenzie R subcatchment). These three locations coincide with instances of haplotype sharing across the Burdekin–Fitzroy boundary and have adjacent headwaters with the Burdekin, implying contemporary dispersal across a long-term barrier. Independent analysis of the southern cluster revealed further genetic subdivision into three distinct clusters of neighboring drainages (highest delta *K* for *K *=* *3; Fig. 5B), corresponding to the Mary–Burnett–Kolan drainages, the Fitzroy drainage, and the Pioneer–Proserpine drainages. When investigating individual assignments within the southern cluster beyond the best *K*, we found individual drainages became increasingly differentiated at higher values of *K*, up to *K *=* *6 (Fig. 5B) when all drainages were differentiated and beyond which no further substructure was evident. There was no evidence for a divergent third lineage within the upstream Burnett R as for mtDNA. Three distinct clusters also best described subdivision within the northern cluster (highest delta *K* for *K *=* *3; Fig. 5C), differentiating between the Burdekin–Herbert–Alligator Ck drainages, the Mulgrave–Russell drainage, and the Normanby drainage. Higher values of *K* (Fig. 5C) further differentiated individual drainages, up to *K *=* *4 (Fig. 5C), when all drainages were differentiated except two Herbert R individuals, which showed mixed ancestry between the nearby Burdekin and Alligator Ck drainages. No further substructure was evident.

### Recent migration

Five replicate BAYESASS runs produced highly consistent results and very low estimates of *m* for both the northern and southern sample groups, being 0.0033 or 0.0034 per run (SD 0.0019 per run). However, because *m* represents the fraction of migrants in a population, and sample size for the northern lineage was roughly half that of the southern lineage, migration has been greater southwards across the Burdekin–Fitzroy drainage divide (from the northern lineage into the southern lineage). Individual posterior probabilities of migrant ancestry identified seven individuals as potential migrants (nonmigrant probability < 0.90). All had second-generation migrant probabilities between 0.14 and 0.97 (first-generation migrant probabilities were negligible) and were sampled from headwater locations adjacent the Burdekin–Fitzroy drainage divide. Northern lineage migrants were sampled from Theresa Ck (Fitzroy drainage, *n* = 4), and the Pioneer and Proserpine drainages (*n* = 1 each), while a single southern lineage migrant was sampled from the Burdekin's Suttor R. Locations of cross-drainage dispersal are the same as those identified from the mtDNA haplotype network and microsatellite clustering analyses, although only the Suttor R individual exhibited an mtDNA haplotype from the opposite lineage.

### Divergence date estimation among northern and southern lineages

Coalescent-based analyses in BEAST estimate northern and southern *E*. *m*. *krefftii* mitochondrial lineages originated (diverged) approximately 4.73 million years ago (Ma), in the early Pliocene (95% higher posterior density, HPD: 2.08–8.16 Ma). Earliest divergence within the southern lineage was estimated at 1.28 Ma (95% HPD: 0.54–2.20 Ma), and for the northern lineage 0.74 Ma (95% HPD: 0.22–1.42 Ma), both within the Pleistocene.

### Historical demography

**Figure 4:**
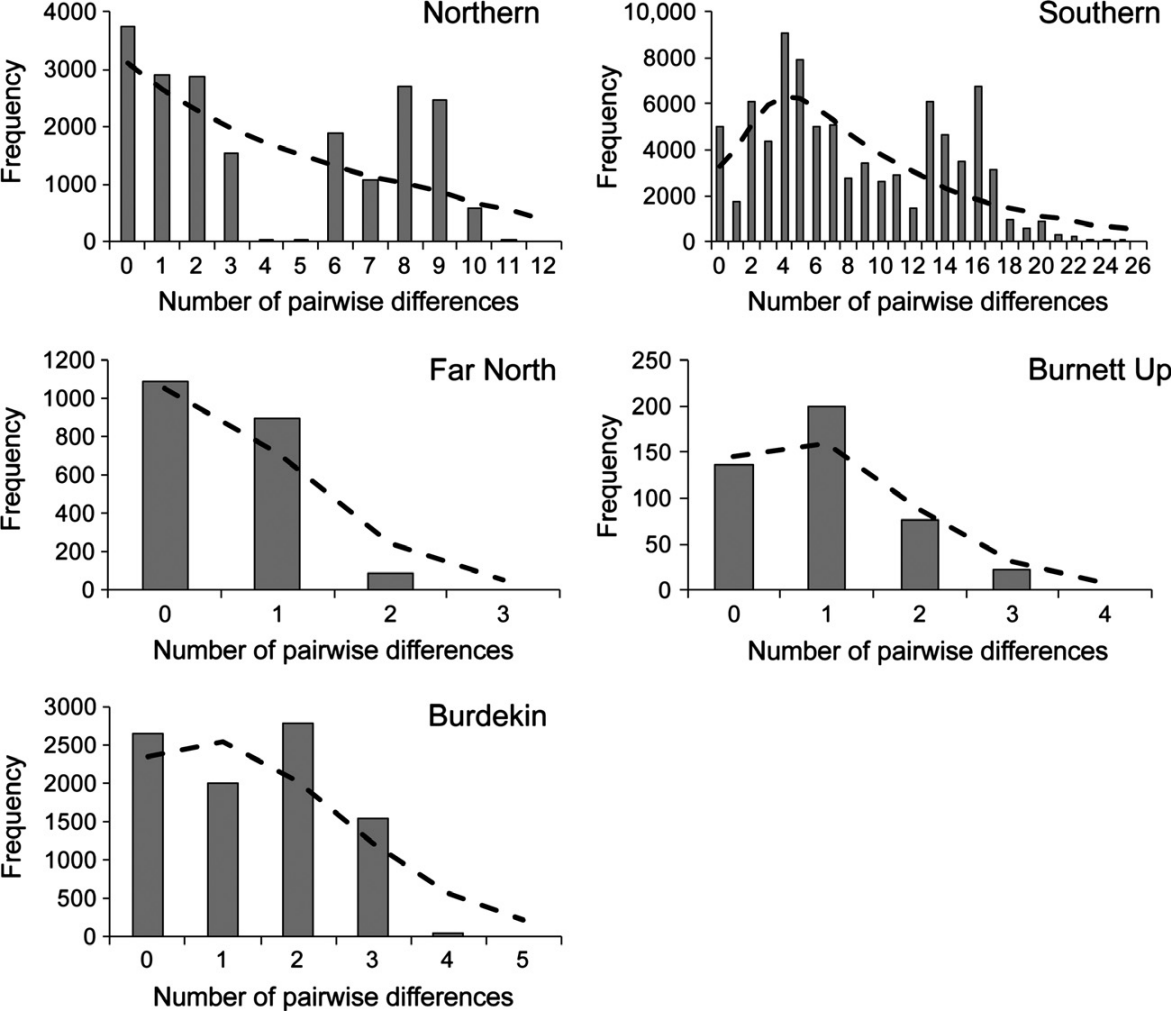
Mismatch distribution among mitochondrial DNA haplotypes for major *Emydura macquarii krefftii* lineages in Northern, Southern and Burnett upstream locations. Northern lineage is further subdivided into Far North and Burdekin lineages. Gray bars show the observed values, and black dashed lines indicate the expected distribution based on a model of sudden demographic expansion.

There was no consistent support for a model of demographic expansion in *E*. *m*. *krefftii*. Estimates of demographic parameters and neutrality tests for major mtDNA clades are presented in Table 2. Although values for *D* and *F*_S_ were negative for most lineages, suggesting population expansion, only the *D* statistic in the Far North clade was significant. However, significant *r* and SSD values indicated a sudden expansion model could not be conclusively rejected in any case (Table 2). Smooth mismatch distributions characterized the upstream Burnett clade and the Burdekin and Far North subclades, indicating demographic expansion. However, in each of these cases, the number of haplotypes was very small, potentially limiting the power of the test. A more ragged distribution was observed for the southern clade, indicating stable demography (Fig. 4). Analysis of the northern clade produced a bimodal distribution, indicating at two distinct sublineages (i.e., Burdekin and Far North subclades; Fig. 4).

Heterozygosity excess tests based on microsatellite data were nonsignificant in all cases, providing no evidence for recent genetic bottlenecks within any sample group at either regional or intraregional scales.

More sophisticated coalescent-based analyses were also performed, making use of both mtDNA and microsatellite data. These included Bayesian skyline plots to estimate changes in effective population size through time, and approximate Bayesian computation (ABC) analyses to differentiate between alternative scenarios of demographic change. However, preliminary results from both methods indicated the current dataset lacks resolution for estimating historical demographic parameters, and results are not presented here.

## Discussion

Australia presents a unique opportunity to study the impact of episodic Pleistocene aridity on current biodiversity in the absence of widespread glaciation. In the present study, fine-scale geographic sampling and analyses of mtDNA and nuclear microsatellites were used to describe genetic structure and diversity in the widespread Krefft's river turtle, *E*. *m*. *krefftii*. Three divergent mtDNA clades were identified within *E*. *m*. *krefftii,* and a strongly hierarchical population structure was evident across its extensive range. Data enabled differentiation between competing biogeographic (arid corridor vs. drainage divide) and demographic (local persistence vs. northwards range expansion) hypotheses related to the impact of cyclic aridity on the east Australian freshwater fauna. These hypotheses are addressed in detail below in relation to the current genetic data.

### Biogeographic barriers: drainage divide or arid corridor?

**Figure 5:**
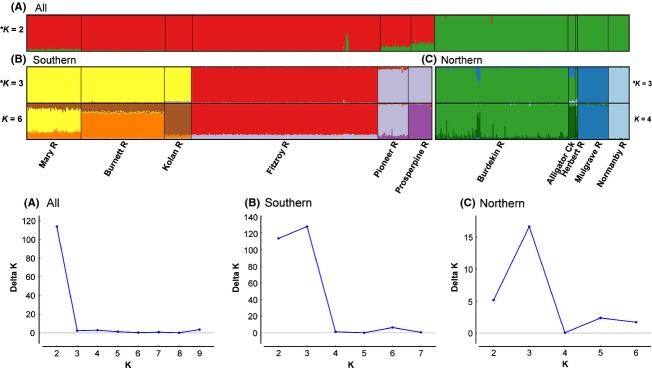
Bayesian population assignment plots (top) for *Emydura macquarii krefftii* individuals sampled from 11 river basins, based on STRUCTURE analyses of 12 microsatellite loci. Individuals are represented by colored vertical bars indicating their percentage genetic membership (*y* axis) within *N* genetic units (*K*). Black lines separate individuals sampled from different drainages (*x* axis). *Indicates the most likely number of clusters (as per Evanno's delta log method, bottom): (A) for the full dataset, indicating primary division between southern and northern genetic clusters; and for the southern (B) and northern (C) clusters analyzed individually, indicating further substructure within regions. Highest level of substructure observed within southern and northern datasets are also indicated, showing differentiation among individual drainage basins.

The most striking pattern in the data was the genetic dichotomy between northern and southern *E*. *m*. *krefftii* lineages in the vicinity of the Burdekin drainage. The disjunction is geographically congruent with genetic “breaks” observed in codistributed taxa, hypothesized to reflect a terrestrial climatic barrier termed the Burdekin Gap. Fine-scale sampling of *E*. *m*. *krefftii* throughout the Burdekin and adjacent catchments, however, revealed the genetic disjunction to be inconsistent with this hypothesis. Turtle populations sampled within the Burdekin drainage but separated by the Gap were closely related genetically at mtDNA and microsatellite data. Low, but significant, *F*_ST_ values among Burdekin locations for microsatellites (range 0.011–0.016) suggest only weak restrictions on dispersal, consistent with distance, a large waterfall (the Burdekin Falls, Fig. 2), and seasonal habitat isolation due to aridity. Greatest genetic divergence in *E*. *m*. *krefftii* occurred across the Burdekin–Fitzroy drainage divide (Figs. 3, 5), among populations just 10's of km apart (Fig. 2). The Burdekin (130,400 km^2^) and Fitzroy (141,100 km^2^) basins are by far the largest and oldest systems on Australia's eastern seaboard, having expanded westwards during the Palaeogene (Jones 2006). Bathymetry indicates they also maintained independent palaeochannel trajectories during glacial low stands, when coastlines extended ∼160 km offshore relative to today (Fielding et al. 2003; Ryan et al. 2007). In past studies, unavailability of samples from within the Burdekin drainage precluded differentiating between these alternative biogeographic hypotheses, yet the drainage divide hypothesis would explain north-south divergence observed in other freshwater taxa. Greatest divergence also occurs across this boundary for east Australian snapping turtles (genus *Elseya*; estimated at *c*. 5.82 Ma, 95% HPD 4.02–7.86 Ma), while populations either side of the Burdekin Gap are distinct only at the intraspecific level within *E*. *irwini* (Todd et al. 2013b). Our data reveal the ancient Burdekin–Fitzroy drainage divide as a novel, long-standing biogeographic barrier disrupting north-south dispersal for freshwater fauna along Australia's eastern coastline. The Burdekin Gap appears comparatively insignificant as a long-term freshwater dispersal barrier, despite its role in structuring terrestrial lineages.

Genetic isolation across watershed boundaries was a pattern observed more generally in *E*. *m*. *krefftii*, while dispersal within drainages appears largely unconstrained. Individual drainage basins typically contained unique haplotypes and pairwise comparisons between drainages at microsatellite data were all highly significant (mean *F*_ST_ 0.209, SD 0.104), with weak or no differentiation among locations within drainages (mean *F*_ST_ 0.008, SD 0.005). Hierarchical relationships among drainages were also evident, especially from analyses in STRUCTURE (Fig. 5). In the north, Burdekin populations are closely related to those in the neighboring Alligator Ck and Herbert drainages, while the Mulgrave–Russell and Normanby populations are distinct. In the south, Fitzroy populations are distinct, while those from the Burnett, Mary and Kolan Rivers are closely related, as are those from the Pioneer and Proserpine Rivers. Similar interdrainage relationships are reported for codistributed freshwater taxa (McGlashan and Hughes 2001; Thacker et al. 2007; Jerry 2008) and indicate a common biogeographic history reflecting long-term patterns of drainage interconnectivity. For example, genetic similarity between Burnett and Mary populations of the locally endemic turtle *Elseya albagula* (Burnett, Mary and Fitzroy Rivers) has been linked to geological evidence for a recent confluence of these river systems, severed by rising sea levels only ∼12,000 years ago (Todd et al. 2013a).

Evidence for contemporary dispersal between divergent northern and southern clades indicates the Burdekin–Fitzroy drainage boundary is not a static barrier and that watershed boundaries are occasionally overcome by overland dispersal. Instances of haplotype sharing coincide geographically with three clear regions of admixture uncovered in STRUCTURE analyses and with the sampling locations of second-generation migrants identified in BYAESASS analyses of microsatellite data, indicating dispersal across adjacent headwaters (Burdekin Bowen R and the Pioneer–Proserpine Rivers, and the Burdekin Suttor R and Fitzroy Theresa Ck). Closer relatedness of one Burdekin haplotype, found in six individuals sampled from the Suttor R close to the Fitzroy boundary, to Fitzroy haplotypes may also indicate historical crossing of the drainage divide. Despite the limited haplotype sharing among drainages, complex relationships among southern haplotypes also imply periodic cross-drainage dispersal followed by isolation and divergence. Isolation by drainage divides coupled with occasional cross-drainage dispersal may be important in generating considerable genetic diversity observed across the whole *E*. *m*. *krefftii* distribution.

### Historical demography: local persistence or northwards expansion?

The Burdekin lowlands experienced severe local aridity during glacial maxima relative to neighboring regions,consistent with the Burdekin's current low species diversity. Early Pliocene fossil deposits from the north-west Burdekin (Fig. 2; 3.62 ± 0.05 Ma, Mackness et al. 2000) of water birds and turtles, including riverine specialist *Elseya* sp., swamp-adapted *Chelodina* sp., and *Emydura* indistinguishable from living *E*. *m*. *krefftii*, indicate diverse aquatic habitat here before establishment of Pleistocene cyclic aridity (Thomson and Mackness 1999). We evaluated alternative possibilities that extant populations of Krefft's turtles in this region are a surviving lineage that persisted despite periodic local climatic harshness, or represent recent northwards expansion from climatically more stable southern habitats. The degree of divergence observed between northern and southern *E*. *m*. *krefftii* lineages supports the former hypothesis and rules out recent northwards expansion of a southern lineage. Coalescent analyses in BEAST estimate their divergence at *c*. 4.73 Ma, and confidence intervals (95% HDP 2.08–8.16 Ma) predate evidence for widespread aridity in eastern Australia around 1.5 Ma (Martin 2006; McLaren and Wallace 2010). Extant northern Krefft's populations may indeed be descendants of a surviving lineage that persisted despite locally severe Pleistocene aridity.

Stark differences observed in levels of genetic diversity between northern and southern Krefft's lineages may reflect regional differences in historical demography and climatic regime. For the southern lineage, high genetic diversity and evidence of stable demographic history are consistent with current high freshwater species diversity in the Fitzroy, Burnett and Mary Rivers region. Concentration of freshwater turtles (seven species, three local endemics) and other relict Gondwanan lineages (e.g., lungfish and saratoga), especially, implies enduring aquatic habitat (Todd et al. 2013a). By contrast, genetic diversity estimates for northern *E*. *m*. *krefftii* were strikingly low. If northern Krefft's populations persisted despite local aridity, as our data suggest, recurrent population bottlenecks may be expected, followed by demographic expansions as favorable conditions returned. However, we found only weak support for a demographic expansion model in northern *E*. *m*. *krefftii*. We were unable to rigorously test hypotheses related to historical bottlenecks, owing to low haplotypic diversity and resolution of the current dataset for estimating historical demographic parameters. It may be that current low genetic diversity in northern *E*. *m*. *krefftii* results from increased genetic drift relative to southern populations, due to local habitat instability driving extinction-recolonization dynamics and temporal fluctuations in effective population size.

Many Northern Hemisphere species, including freshwater fishes and turtles, show clear signatures of genetic bottlenecks and range reductions into isolated glacial refugia, followed by dramatic demographic and range expansion since the end of the last ice age (Starkey et al. 2003; Seifertova et al. 2012). Cyclic Pleistocene aridity in Australia appears not to have produced molecular signatures of wide-scale contraction to major refugia and subsequent macrogeographical range expansion as seen for temperate Northern Hemisphere biotas (Hewitt 2000). Recent work in Australia's arid zone suggests a less overwhelming and more idiosyncratic response of taxa, whereby divergence builds up over repeated cycles of localized contraction and expansion from patchwork refugia throughout a species range (Byrne 2008; Lanier et al. 2013). In the case of *E*. *m*. *krefftii*, a generalist ecology may have facilitated survival in multiple peripheral habitats (e.g., isolated waterholes and spring-fed streams), and swift recolonization once freshwater connections re-established. An ability to move overland and abandon drying habitat in search of permanent waterbodies potentially further facilitated their survival during periods of climatic harshness in the Burdekin region, when many other freshwater taxa clearly were extirpated.

### A third divergent matrilineage in *E. m. krefftii*

A third divergent *E. m*. *krefftii* matrilineage was found isolated to an upstream location within the Burnett drainage, but was not supported by nuclear microsatellite data. Although such incongruence between nuclear and mitochondrial datasets may result from male-biased gene flow and/or female philopatry to nesting sites (Toews and Brelsford 2012), reported for freshwater turtles elsewhere (Sheridan et al. 2010), neither can sufficiently explain the observed concentration of highly divergent haplotypes within this single upstream location. Similarly, although all third lineage individuals were collected within impounded waters of the Barambah Dam, multiple man-made impoundments exist throughout the subspecies' range, and none are old enough to cause the observed divergence in a long-lived species. *Emydura macquarii* is popular in the pet trade. Therefore, a plausible explanation involves anthropogenic translocation of turtles into Barambah Dam from elsewhere. Introgression could have increased the local frequency of a divergent *E*. *macquarii* mitochondrial lineage within the impoundment, itself preventing divergent haplotypes from being dispersed throughout the catchment. Our analysis of mtDNA from related *E*. *macquarii* subspecies did not reveal a likely source population, although our sampling was geographically restricted. Broader geographic sampling is necessary to confirm a translocated origin of the divergent 3rd lineage within *E*. *m*. *krefftii*.

### Subspecies relationships

Chelonian subspecies designations often reflect subtle morphological variation among nonoverlapping geographic entities, and it is not uncommon for these to be unsupported by molecular data (e.g., Fritz et al. 2008, 2012). Genetic distinctiveness of the four *E. macquarii* subspecies, as currently recognized, was not supported by our mtDNA data, corroborating earlier allozyme work finding no fixed differences among them (Georges and Adams 1996). Within *E*. *m*. *krefftii*, divergence between northern and southern clades (2.23% uncorrected ND4 *p*-distance) is within the range typically observed among congeneric chelonian species (range 1.5–18.3%; Fritz et al. 2012). Despite restricted geographical sampling of the remaining three subspecies, our haplotype network shows *E*. *m*. *macquarii* and *E*. *m*. *nigra* haplotypes are nested within the southern *E*. *m*. *krefftii* lineage (Fig. 3). Only *E*. *m*. *emmotti* appears distinct genetically.

Range-wide investigation of morphological and genetic variation in *E. macquarii* using both mitochondrial and sensitive nuclear markers (e.g., microsatellites or SNPs) is warranted. Such an analysis may prove especially revealing, as *E*. *macquarii* is one of few very widespread species inhabiting south-eastern Australia shown to be genetically structured. Although biogeographically complex and relatively well-studied, historical relationships and dispersal patterns among major freshwater biogeographic regions in this area remain poorly resolved (Unmack 2001; Thacker et al. 2007; Jerry 2008; Bentley et al. 2010; Faulks et al. 2010; Unmack and Dowling 2010).

## Conclusions

Range-wide genetic structure and molecular diversity in a widespread freshwater turtle, *E*. *m*. *krefftii*, primarily reflects isolation by drainage divides, with evidence of occasional overland dispersal. We found little evidence that an arid corridor is responsible for structuring Krefft's turtles along Australia's east coast, and newly identify a significant biogeographic barrier in the form of an ancient drainage divide between the geographically extensive Burdekin and Fitzroy basins. Although *E*. *m*. *krefftii* apparently persisted despite severe local aridity associated with the Burdekin Gap through multiple glacial cycles, habitat instability and increased genetic drift may be responsible for very low contemporary genetic diversity compared with southern populations. Our study highlights the importance of fine-scale geographical sampling in evaluating the role of biogeographic barriers and demonstrates the utility of integrating mitochondrial sequences and sensitive nuclear datasets for inferring evolutionary history.

The broader *E. macquarii* complex may be a valuable model taxon for unraveling biogeographic complexity in south-eastern Australia for freshwater taxa, and may help to answer several long-standing questions regarding historical patterns of freshwater connectivity and dispersal in this region.

## Data Accessibility

Sequences are available at GenBank under accessions KF181795-KF181853 for control region and KF181854–KF181885 for *ND4*.

## Supporting Information

**Appendix S1.** Specimens examined.

**Appendix S2.** Mitochondrial DNA haplotype frequencies.
