# Supplementary material for: Influence of drainage divides versus arid corridors on genetic structure and demography of a widespread freshwater turtle, Emydura macquarii krefftii, from Australia
Source: Ecol Evol. 2014 Feb 11;4(5):606–22. doi: 10.1002/ece3.968 (PMC4098141; doi:10.1002/ece3.968)
Supplement: Supplementary file 1 — Appendix S1. Specimens examined. [file ece30004-0606-sd1.docx]

**Appendix S1: Electronic Supporting Information**

***Specimens examined***

Table S1. Sampling locality information for specimens used in genetic analyses of *Emydura macquarii krefftii* and related subspecies.

| **Drainage**  Sub-region | **Locality** | **Latitude** | **Longitude** | ***N*** |
| --- | --- | --- | --- | --- |
| *Emydura* *m*. *krefftii* |  |  |  |  |
| **Normanby** |  |  |  |  |
| Normanby R | Jack’s Lake | -14.877 | 144.433 | 6 |
| Normanby R | Leichardt Hole | -15.259 | 144.613 | 1 |
| Laura R | Crocodile Hole | -15.653 | 144.595 | 15 |
| **Mulgrave-Russell** |  |  |  |  |
| Alice R | Eubenangee Swamp | -17.409 | 145.982 | 33 |
| **Herbert** |  |  |  |  |
| Herbert R | Jourama Falls | -18.867 | 146.125 | 3 |
| **Alligator Ck** |  |  |  |  |
| Alligator Ck | Downstream pool | -19.309 | 146.765 | 8 |
| **Burdekin** |  |  |  |  |
| Burdekin R | Greenvale Station | -19.083 | 145.050 | 32 |
| Bowen R | Urannah Causeway | -20.914 | 148.427 | 6 |
| Bowen R | Cloverly Pool | -20.921 | 148.327 | 5 |
| Bowen R | Eungella Dam | -21.145 | 148.385 | 32 |
| Suttor R | Kennedy Billabong | -21.417 | 147.677 | 68 |
| Suttor R | Mistake Cr, Laglan Rd crossing | -22.564 | 147.073 | 32 |
| **Pioneer** |  |  |  |  |
| Pioneer R | Mirani bridge | -21.159 | 148.860 | 33 |
| **Proserpine** |  |  |  |  |
| Proserpine R | Below Proserpine dam | -20.366 | 148.392 | 25 |
| **Fitzroy** |  |  |  |  |
| Fitzroy R | Alligator Ck Junction | -23.190 | 150.402 | 32 |
| Fitzroy R | Marlborough Ck | -22.969 | 149.867 | 36 |
| Mackenzie R | Nogoa R, Fairburn Dam | -23.660 | 148.077 | 32 |
| Mackenzie R | Nogoa R, Van Dyke Ck | -24.074 | 147.761 | 17 |
| Mackenzie R | Carnarvon Ck, Warrinilla Station | -24.924 | 148.600 | 16 |
| Mackenzie R | Comet R, Carnarvon Ck | -25.060 | 148.230 | 1 |
| Mackenzie R | Theresa Ck, Hoods Lagoon | -22.818 | 147.642 | 6 |
| Dawson R | Korcha pump house | -25.44 | 148.6642 | 4 |
| Dawson R | Hutton Ck, Warndoo | -25.77 | 148.7363 | 8 |
| Dawson R | Moura boat ramp | -24.603 | 149.913 | 23 |
| Dawson R | Glebe Weir | -25.467 | 150.028 | 26 |
| **Burnett** |  |  |  |  |
| Upstream | Barambah Dam | -26.305 | 151.979 | 32 |
| Midstream | Wharton Weir, Gayndah | -25.615 | 151.592 | 28 |
| Downstream | Wallaville Weir, Berrembea | -25.051 | 152.099 | 30 |
| **Mary** |  |  |  |  |
| Upstream | Borumba Dam | -26.526 | 152.565 | 27 |
| Upstream | Kenilworth | -26.587 | 152.730 | 7 |
| Downstream | Banting | -25.627 | 152.610 | 12 |
| Downstream | Pioneer’s Rest | -25.700 | 152.573 | 5 |
| Downstream | Petrie Park, Tiaro boat ramp | -25.718 | 152.576 | 7 |
| **Kolan** |  |  |  |  |
| Kolan R | Smith’s Crossing | -24.816 | 152.166 | 29 |
|  |  |  |  |  |
| *Emydura* *m*. *emmotti* |  |  |  |  |
| **Cooper Ck** | Cullyamurra Waterhole | -27.701 | 140.843 | 5 |
|  |  |  |  |  |
| *Emydura* *m*. *macquarii* |  |  |  |  |
| **Murray-Darling** | Murray R, Doctors Point Lagoon | -36.097 | 146.934 | 5 |
|  |  |  |  |  |
| *Emydura* *m*. *nigra* |  |  |  |  |
| **Frazer Is** | Lake Birrabeen | -25.507 | 153.063 | 5 |
