# Supplementary material for: Influence of drainage divides versus arid corridors on genetic structure and demography of a widespread freshwater turtle, Emydura macquarii krefftii, from Australia
Source: Ecol Evol. 2014 Feb 11;4(5):606–22. doi: 10.1002/ece3.968 (PMC4098141; doi:10.1002/ece3.968)
Supplement: Supplementary file 2 — Appendix S2. Mitochondrial DNA haplotype frequencies. [file ece30004-0606-sd2.docx]

**Appendix S2: Online Supporting Information**

***Haplotype frequencies***

Table S2 Frequencies of control region and ND4 mtDNA haplotypes for *Emydura macquarii krefftii* by drainage basin

| **Haplotype^1^** | **Location and sample size** | | | | | | | | | | |
| --- | --- | --- | --- | --- | --- | --- | --- | --- | --- | --- | --- |
|  | Mary  (57) | Burnett  (90) | Kolan  (29) | Fitzroy  (206) | Pioneer  (33) | Proserpine  (25) | Burdekin  (143) | Alligator Ck  (8) | Herbert  (3) | Mulgrave-Russell  (33) | Normanby  (22) |
| *CR* |  |  |  |  |  |  |  |  |  |  |  |
| 1 | 39 | - | - | - | - | - | - | - | - | - | - |
| 2 | 6 | - | - | - | - | - | - | - | - | - | - |
| 3 | 9 | - | - | - | - | - | - | - | - | - | - |
| 4 | 2 | - | - | - | - | - | - | - | - | - | - |
| 5 | 1 | - | - | - | - | - | - | - | - | - | - |
| 6 | - | 35 | - | 3 | - | - | - | - | - | - | - |
| 7 | - | 3 | - | - | - | - | - | - | - | - | - |
| 8 | - | 1 | - | - | - | - | - | - | - | - | - |
| 9 | - | 11 | - | - | - | - | - | - | - | - | - |
| 10 | - | 4 | 5 | - | - | - | - | - | - | - | - |
| 11 | - | 1 | - | - | - | - | - | - | - | - | - |
| 12 | - | 1 | - | - | - | - | - | - | - | - | - |
| 13 | - | 2 | - | - | - | - | - | - | - | - | - |
| 14 | - | 18 | - | - | - | - | - | - | - | - | - |
| 15 | - | 3 | - | - | - | - | - | - | - | - | - |
| 16 | - | 8 | - | - | - | - | - | - | - | - | - |
| 17 | - | 1 | - | - | - | - | - | - | - | - | - |
| 18 | - | 2 | - | - | - | - | - | - | - | - | - |
| 19 | - | - | 6 | - | - | - | - | - | - | - | - |
| 20 | - | - | 5 | - | - | - | - | - | - | - | - |
| 21 | - | - | 1 | - | - | - | - | - | - | - | - |
| 22 | - | - | 9 | - | - | - | - | - | - | - | - |
| 23 | - | - | 3 | - | - | - | - | - | - | - | - |
| 24 | - | - | - | 18 | - | - | - | - | - | - | - |
| 25 | - | - | - | 33 | - | - | 1 | - | - | - | - |
| 26 | - | - | - | 78 | - | - | - | - | - | - | - |
| 27 | - | - | - | 1 | - | - | - | - | - | - | - |
| 28 | - | - | - | 14 | - | - | - | - | - | - | - |
| 29 | - | - | - | 11 | - | - | - | - | - | - | - |
| 30 | - | - | - | 9 | - | - | - | - | - | - | - |
| 31 | - | - | - | 2 | - | - | - | - | - | - | - |
| 32 | - | - | - | 1 | - | - | - | - | - | - | - |
| 33 | - | - | - | 1 | - | - | - | - | - | - | - |
| 34 | - | - | - | 5 | - | - | - | - | - | - | - |
| 35 | - | - | - | 4 | - | - | - | - | - | - | - |
| 36 | - | - | - | 1 | - | - | - | - | - | - | - |
| 37 | - | - | - | 3 | - | - | - | - | - | - | - |
| 38 | - | - | - | 14 | - | - | - | - | - | - | - |
| 39 | - | - | - | 2 | - | - | - | - | - | - | - |
| 40 | - | - | - | 1 | - | - | - | - | - | - | - |
| 41 | - | - | - | 1 | - | - | - | - | - | - | - |
| 42 | - | - | - | 3 | - | - | - | - | - | - | - |
| 43 | - | - | - | - | 33 | 24 | 1 | - | - | - | - |
| 44 | - | - | - | - | - | 1 | - | - | - | - | - |
| 45 | - | - | - | - | - | - | 42 | - | - | - | - |
| 46 | - | - | - | 1 | - | - | 89 | - | 1 | - | - |
| 47 | - | - | - | - | - | - | 1 | - | - | - | - |
| 48 | - | - | - | - | - | - | 1 | - | - | - | - |
| 49 | - | - | - | - | - | - | 1 | - | - | - | - |
| 50 | - | - | - | - | - | - | 6 | - | - | - | - |
| 51 | - | - | - | - | - | - | 1 | - | - | - | - |
| 52 | - | - | - | - | - | - | - | 8 | 2 | 32 | 3 |
| 53 | - | - | - | - | - | - | - | - | - | 1 | - |
| 54 | - | - | - | - | - | - | - | - | - | - | 4 |
| 55 | - | - | - | - | - | - | - | - | - | - | 14 |
| 56 | - | - | - | - | - | - | - | - | - | - | 1 |
| *ND4* |  |  |  |  |  |  |  |  |  |  |  |
| 1 | 1 | 45 | 25 | 57 | - | - | - | - | - | - | - |
| 2 | 40 | 3 | - | - | - | - | - | - | - | - | - |
| 3 | 7 | - | - | - | - | - | - | - | - | - | - |
| 4 | 8 | - | - | - | - | - | - | - | - | - | - |
| 5 | 2 | - | - | - | - | - | - | - | - | - | - |
| 6 | - | 12 | - | - | - | - | - | - | - | - | - |
| 7 | - | 26 | - | - | - | - | - | - | - | - | - |
| 8 | - | 3 | - | - | - | - | - | - | - | - | - |
| 9 | - | 1 | - | - | - | - | - | - | - | - | - |
| 10 | - | - | 4 | - | - | - | - | - | - | - | - |
| 11 | - | - | - | 33 | - | - | 1 | - | - | - | - |
| 12 | - | - | - | 25 | - | - | - | - | - | - | - |
| 13 | - | - | - | 10 | - | - | - | - | - | - | - |
| 14 | - | - | - | 15 | - | - | - | - | - | - | - |
| 15 | - | - | - | 2 | - | - | - | - | - | - | - |
| 16 | - | - | - | 43 | - | - | - | - | - | - | - |
| 17 | - | - | - | 1 | - | - | - | - | - | - | - |
| 18 | - | - | - | 4 | - | - | - | - | - | - | - |
| 19 | - | - | - | 1 | - | - | - | - | - | - | - |
| 20 | - | - | - | 2 | - | - | - | - | - | - | - |
| 21 | - | - | - | 1 | - | - | - | - | - | - | - |
| 22 | - | - | - | 1 | - | - | - | - | - | - | - |
| 23 | - | - | - | 7 | 33 | 25 | 1 | - | - | - | - |
| 24 | - | - | - | 1 | - | - | 101 | - | 1 | - | - |
| 25 | - | - | - | - | - | - | 1 | - | - | - | - |
| 26 | - | - | - | - | - | - | 6 | - | - | - | - |
| 27 | - | - | - | - | - | - | 17 | - | - | - | - |
| 28 | - | - | - | - | - | - | 16 | - | - | - | - |
| 29 | - | - | - | - | - | - | - | 8 | 2 | 33 | 22 |

**^1^**Haplotypes represent 439 bp control region (CR) and 670 bp NADH dehydrogenase subunit 4 (ND4), together with 70 bp tRNAHis, 63 bp tRNASer and the first 34 bp tRNALeu (GenBank Accession numbers: KF181795-KF181850, KF181854-KF181882).
